# Zeolite and Chitosan: A sustainable duo to improve sesame drought resistance

**DOI:** 10.1371/journal.pone.0340215

**Published:** 2026-02-17

**Authors:** Amir Mohammad Abedi, Ali Heidarzadeh, Seyed Ali Mohammad Modarres-Sanavy

**Affiliations:** Department of Agronomy, Faculty of Agriculture, Tarbiat Modares University, Tehran, Iran; Institute for Biological Research, University of Belgrade, SERBIA

## Abstract

Climate change and increasing competition for water in arid and semi-arid regions intensify moisture stress in sesame production. Deficit irrigation (DI) is therefore essential to optimize yield with limited water. This study investigated the potential of two eco-friendly amendments zeolite and chitosan to mitigate drought effects in sesame. A split-factorial experiment was conducted in a randomized complete block design with three replications at two locations (Tehran and Yazd). Irrigation treatments included optimal irrigation (I1; 50% available soil water depletion), moderate drought (I2; 65%), and severe drought (I3; 80%). Subplots received zeolite (0 and 4.5 t ha⁻^1^) and foliar applications of chitosan (0.4% and 0.5%) or controls. Under I1, the combined application of zeolite and 0.5% chitosan markedly improved leaf area index (LAI), grain yield, oil content, and harvest index. Antioxidant enzyme activities (CAT and POD) increased, accompanied by a moderate rise in malondialdehyde (MDA). Under I2, treatments enhanced LAI, grain yield, oil content, and soluble protein without significant changes in MDA. Under I3, zeolite and chitosan improved LAI, grain and oil yield, increased POD and CAT activities, and reduced MDA. Overall, applying zeolite (4.5 t ha⁻^1^) and chitosan (0.5%) effectively alleviated drought stress by enhancing physiological performance, yield, and antioxidant capacity. These results demonstrate the potential of zeolite and chitosan as sustainable solutions to improve drought tolerance in sesame cultivation, particularly in water-scarce environments.

## 1. Introduction

Sesame (*Sesamum indicum* L.) is a drought-tolerant oilseed crop widely cultivated in arid and semi-arid regions due to its adaptability and high oil content, which can reach up to 63%, exceeding major oilseeds such as groundnut, sunflower, rapeseed, and soybean [1]. Its seeds are also rich in proteins, essential fatty acids, vitamins, and minerals, contributing to its medicinal and nutritional value. The oil’s exceptional stability is attributed to antioxidants like sesamin, sesamolin, and sesamol [2].

Although relatively drought-tolerant, sesame is typically grown under rainfed and nutrient-poor conditions, making it vulnerable to terminal drought stress, especially during critical growth stages [3]. Drought restricts growth by causing osmotic stress, limiting photosynthesis, and disturbing biochemical pathways. Reduced CO₂ availability due to stomatal closure, along with oxidative stress and reactive oxygen species (ROS) accumulation, can damage cellular structures and impair crop performance [4,5]. Plants activate antioxidant enzymes like catalase (CAT), superoxide dismutase (SOD), and peroxidase (POD) to cope with ROS-induced damage [6].

Previous studies have shown that drought during flowering significantly reduces sesame yield and oil quality by inducing flower abortion and limiting capsule development [3,7]. Regulated deficit irrigation (RDI) has emerged as a promising technique for optimizing water use efficiency without severely affecting yield. For instance, supplying 50–70% of crop water needs improved water productivity despite reducing grain yield [8]. Climate change is expected to intensify water scarcity, requiring integrated solutions to improve crop resilience [9]. Complementary to RDI, bio-based soil amendments are vital to reducing reliance on chemical fertilizers and preserving environmental health [10].

Among these, chitosan-a biodegradable, non-toxic biopolymer- has shown potential in enhancing plant growth and stress tolerance by improving antioxidant defense and modulating physiological processes such as stomatal closure and hormone signaling [11,12]. Its foliar application improves plant metabolism and productivity under stress conditions. Zeolites, natural aluminosilicate minerals with high water retention and ion exchange capacities, also support plant performance under drought by improving soil moisture and nutrient availability [13,14]. Natural zeolites, particularly clinoptilolite, exhibit a highly porous three-dimensional aluminosilicate framework characterized by high specific surface area (up to >300 m^2^·g⁻^1^ for fine particles), pore volume of 0.2–0.3 cm^3^·g⁻^1^, and uniform microporous channels. This structure confers exceptional reversible water adsorption capacity, enabling the retention of several weight percent of water within the crystalline lattice. Consequently, zeolites are widely recognized as effective soil amendments capable of significantly enhancing soil water-holding capacity, reducing irrigation frequency, preventing nutrient leaching, and providing gradual release of moisture and cations to plant roots [15,16]. Zeolite application has been associated with increased soil water-holding capacity and reduced irrigation demand in arid cropping systems [17]. showing qualitative and quantitative benefits on crops [13,18].

Despite the known benefits of chitosan and zeolite in enhancing plant tolerance to drought stress, there is limited information on their combined effects when integrated with regulated deficit irrigation (RDI) in sesame cultivation. Considering the critical impact of water deficit on morphological traits (such as plant height, leaf area index, and capsule length), biochemical responses (including antioxidant enzyme activities like catalase and peroxidase), and yield components (grain yield, oil content, and oil yield), this study aims to comprehensively evaluate the interactive effects of RDI and foliar applications of chitosan and zeolite on sesame growth, physiological resilience, and productivity. The objective is to identify sustainable management practices that improve drought tolerance, optimize water use efficiency, and maximize sesame yield and oil quality under water-limited conditions.

Despite extensive research on drought-mitigation strategies in various oilseed crops, information remains limited on the integrated use of regulated deficit irrigation (RDI) combined with natural, eco-friendly soil and foliar amendments in sesame (*Sesamum indicum* L.). Although the individual benefits of chitosan and zeolite on plant growth, antioxidant activity, and soil water retention have been documented in several species, their combined application under field-scale deficit irrigation in sesame has not been previously investigated.To the best of our knowledge, this is the first comprehensive field study that simultaneously evaluates the synergistic effects of regulated deficit irrigation, foliar-applied chitosan, and soil-incorporated natural clinoptilolite-rich zeolite on physiological responses, antioxidant defense systems, water-use efficiency, seed yield, oil content, and fatty acid profile of sesame grown in arid and semi-arid regions. This integrated approach aims to maximize water productivity while minimizing reliance on chemical inputs, thereby providing a sustainable, low-cost, and environmentally friendly strategy to enhance climate resilience and maintain both quantitative and qualitative yield traits of sesame under projected water-scarce scenarios induced by climate change.

## 2. Materials and methods

### 2.1. Site description

**Fig 1:**
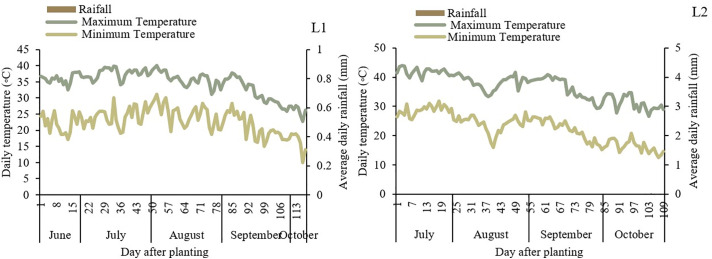
Meteorological data from the Iran Meteorological Organization during the growing season. Tehran (L1) and Yazd (L2).

The experiment was conducted at two different locations (Tehran and Yazd) within a single growing season. Field experiments were conducted during the 2022–2023 growing seasons at two research stations in Iran: Tehran (L1), representing a semi-arid climate, and Yazd (L2), representing an arid climate. The regions receive annual rainfall of approximately 240 mm (L1) and 60 mm (L2). Meteorological data, including temperature and precipitation, were obtained from the Iran Meteorological Organization. Detailed results are in (Fig 1). Soil samples were collected from two depths (0–30 cm and 30–60 cm) prior to planting to assess the physical and chemical properties. Detailed soil data are presented in Table 1.

**Table 1:** Soil physical and chemical properties at both experimental stations.

Depth (cm)	Soil texture	Sand	Silt	Clay	pH	EC	OM	N	P	K	Fe	Zn	Cu
		----------(%)----------				(dS.m^− 1^)	------(%)------		------------------ (mg kg^− 1^) ------------------				
**Tehran (L1)**													
0-30	SL	72	14	14	7.69	0.88	2.13	0.12	180	818	8.2	1.82	2.56
30-60	SL	72	16	12	7.76	0.64	1.68	0.9	184	862	7.3	1.33	2.44
**Yazd (L2)**													
0-30	SL	75	20	5	7.61	1.68	2.20	0.10	41.6	360	7.1	0.91	0.72
30-60	SL	73	18	9	7.81	1.42	1.84	0.94	50.3	384	6.7	0.87	0.71

SL: Sandy loam, OM: Organic matter.

### 2.2. Experimental design

A split factorial experiment based on a randomized complete block design (RCBD) with three replications was implemented. The main plot factor was irrigation regime with three levels: full irrigation at 50% available soil water depletion (ASWD) (I1), moderate deficit at 65% ASWD (I2), and severe deficit at 80% ASWD (I3). The sub-plot included factorial combinations of two levels of zeolite (0 and 4.5 t ha ⁻ ¹) and four levels of foliar spray: 0.4% chitosan (C1), 0.5% chitosan (C2), distilled water as control (C3), and 1% acetic acid (C4).

### 2.3. Treatments

#### 2.3.1. Irrigation regimes.

Irrigation was scheduled based on soil moisture depletion in the root zone using Time-Domain Reflectometry (TDR). Water stress was applied at the flowering stage (BBCH-65), and irrigation was managed based on the percentage of ASWD following the approach described by Behera and Panda [19]. Soil moisture was measured at 0–30 cm and 30–60 cm depths, and gravimetric calibration was performed according to Vanclooster [20]. The maximum allowable depletion (MAD) of soil water was calculated using Equation (1), and irrigation volume (Vd) was determined using Equations (2) and (3):


$$\begin{array}{c} MAD(\%)=\frac{\text{1}}{n}\sum {\mathrm{\backslash nolimits}}_{\text{1}}^{n}\frac{FCi-\theta i}{FCi-PWp}\times \text{1}\text{0}\text{0} \end{array}$$ (1)

ASW=FC−PWP  (2)

$$Vd=\frac{MAD\times ASW\times Rz\times A}{\text{1}\text{0}\text{0}}$$ (3)

where FC is field capacity, θ is soil moisture before irrigation, PWP is permanent wilting point, Rz is root zone depth, A is plot area, and n is number of soil layers. Irrigation was terminated 10 days before harvest in all treatments.

#### 2.3.2. Zeolite application.

**Fig 2:**
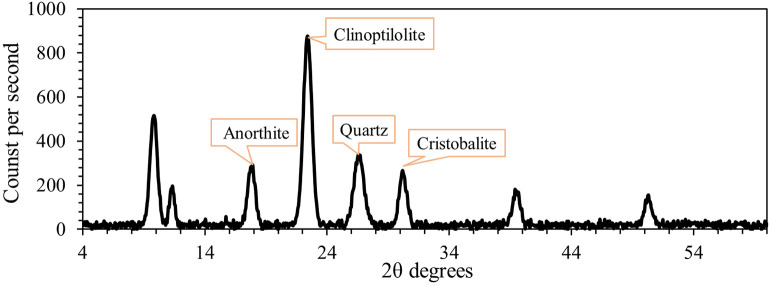
X-ray diffraction (XRD) analysis of mineral phases. Identification of Clinoptilolite, Anorthite, Quartz, and Cristobalite.

Zeolite was applied at a rate of 4.5 tons per hectare during seedbed preparation. It was incorporated into the top 0–50 cm of dry soil before irrigation. The zeolite used was clinoptilolite with a particle size of 400 mesh (<35μm), bulk density of 2.2 g/cm^3^, and cation exchange capacity of 160 meq/100 g. The mineralogical composition and chemical properties of the zeolite are presented in (Fig 2) and Table 2, respectively.

**Table 2:** Type, structure, and chemical properties of the zeolite used in the experiment.

Type of zeolite: Clinoptilolite								
Structure: KNa₂Ca₂(Si₂₉Al₇)O₇₂· 24H₂O								
Element	Ag^+^	Al^3+^	Ba²⁺	Be²⁺	Ca²⁺	Cd²⁺	Mn²⁺	Na⁺
ppm	<0.5	53690	299	2.1	8595	<0.1	12	14684
Element	Co²⁺	Cu²⁺	Fe³⁺	K⁺	Li⁺	Mg²⁺	Mo⁶⁺	Hg⁺
ppm	<1	25	5634	23435	31	4308	0.63	<0.1
Chemical Compounds	SiO₂	Al₂O₃	Fe₂O₃	CaO	Na₂O	P₂O₅	MnO	MgO
%	67.51	12.12	1.32	1.03	1.59	0.029	0.016	0.78

Mineral structure analyzed by XRD, chemical composition by XRF, and elements by MMS-DAM.

#### 2.3.3. Chitosan foliar application.

Chitosan was applied as a foliar spray in two stages: at the onset of flowering (BBCH-65) and during capsule ripening (BBCH-75). To ensure the effect of chitosan, a control treatment (distilled water) and a 1% acetic acid treatment were used as a chitosan solvent.

### 2.4. Trait measurements

#### 2.4.1. Morphological traits and yield components.

Five plants per plot were randomly selected for measuring plant height, capsule length, seeds per capsule, and 1000-seed weight. Leaf Area Index (LAI) was recorded using a portable LAI meter. Grain and biological yields were determined from a 1 m^2^ harvest area after removing border effects. Harvest index was calculated using Equation (4):


$$\text{Harvest index}(\%)=\frac{Grain\;yield}{Biological\;yield}\times \text{1}\text{0}\text{0}$$ (4)

#### 2.4.2. Oil content.

Oil was extracted using Soxhlet apparatus with n-hexane for 10 hours as per Visavadiya [21]. Extracts were dried under vacuum at 40°C using a rotary evaporator, following the protocols of Bozan and Temelli [22] and Carvalho [23].

#### 2.4.3. Malondialdehyde (MDA) content.

MDA was quantified based on Heath and Packer [24]. Leaf tissues (0.5 g) were homogenized in phosphate buffer, centrifuged, and reacted with thiobarbituric acid in trichloroacetic acid. Absorbance was recorded at 532 and 600 nm, and MDA was calculated using an extinction coefficient of 155 mM⁻^1^ cm⁻^1^.

#### 2.4.4. Soluble protein content.

Protein content was determined via the Bradford assay [25]. Plant extracts were reacted with Bradford reagent, and absorbance was measured at 595 nm. A standard curve was used to determine concentration [26].

#### 2.4.5. Catalase activity.

Catalase activity was determined following Türkan [27] with standardized assay conditions. The reaction mixture contained 2.6 mL of 80 mM potassium phosphate buffer (pH 7.0) with 1 mM EDTA, 0.4 mL of 3% H₂O₂, and 40 µL of enzyme extract The assay was performed at 25°C using a cuvette with a 1.00 cm pathlength, and the decrease in absorbance at 240 nm was recorded for 60 s. The concentration of decomposed H₂O₂ was calculated using the molar extinction coefficient ε = 39.4 mM⁻^1^ cm⁻^1^, and enzyme activity was expressed as µmol H₂O₂ decomposed min⁻^1^ mg⁻^1^ protein, where 1 unit (U) corresponds to the decomposition of 1 µmol of H₂O₂ per minute under the assay conditions.

#### 2.4.6. Peroxidase activity.

Peroxidase activity was assayed according to Polle [28] under standardized analytical conditions. The reaction mixture contained 3000 µL of 50 mM phosphate buffer (pH 7.0), 10 µL of 3% H₂O₂, 3 µL of 200 mM guaiacol, and 10 µL of enzyme extract (total volume = 3.023 mL). Measurements were conducted at 25°C in a cuvette with a 1.00 cm pathlength, and the increase in absorbance at 470 nm was recorded every 10 s for 60 s. The formation of tetraguaiacol was quantified using the molar extinction coefficient ε = 26.6 mM⁻^1^ cm⁻^1^, and POD activity was expressed as µmol guaiacol oxidized min⁻^1^ mg⁻^1^protein, defining 1 unit (U) as the oxidation of 1 µmol of guaiacol per minute under the given assay conditions.

### 2.5. Statistical analysis

Data from L1 and L2 were analyzed separately due to non-homogeneity of variances (Bartlett’s test). ANOVA was conducted using the GLM procedure in SAS 9.4, and treatment means were compared by LSD at *P* ≤ 0.05. Pearson’s correlation analysis was performed to evaluate relationships among traits.

## 3. Results

To facilitate interpretation, treatment abbreviations used throughout the results are as follows: L1 and L2 represent the experimental locations in Tehran and Yazd, respectively. Irrigation treatments include I1 (50% ASWD), I2 (65% ASWD), and I3 (80% ASWD). Zeolite treatments include Z1 (no application) and Z2 (4.5 t ha⁻^1^ application). Chitosan treatments are designated as C1 (0.4% chitosan), C2 (0.5% chitosan), C3 (distilled water, control), and C4 (1% acetic acid).

### 3.1. Morphological traits

#### 3.1.1. Plant height.

At location L1, irrigation regimes significantly affected plant height. The greatest height (101.60 cm) was observed under optimal irrigation (I1), followed by moderate (I2; 87.99 cm) and severe water deficit (I3; 68.11 cm) Table 3. Application of zeolite at 4.5 t ha ⁻ ¹ (Z2) increased plant height (92.56 cm) compared to the control without zeolite (Z1; 79.56 cm). Chitosan treatments had no significant effect on height. Interaction between irrigation and zeolite showed that Z2 improved plant height under all irrigation levels: 18.85% under I1, 5.94% under I2, and 13.05% under I3 Table 3.

**Table 3:** Mean comparison main effects of irrigation regimes (I), zeolite (Z), and chitosan (C), and two-way interactions of Z*C, I*Z on sesame traits in Tehran (L1).

Treatments	Height (cm)	Capsule length	Biological yield	Oil content	Oil yield
		(cm)	(kg ha^-1^)	(%)	(kg ha^-1^)
**Irrigation regimes**					
I1	101.60 ± 2.88a	2.96 ± 0.08a	8930.4 ± 290.07a	49.60 ± 0.90a	628.93 ± 23.27a
I2	87.99 ± 2.38b	2.49 ± 0.05b	6227.7 ± 300.75b	44.14 ± 1.24b	370.31 ± 17.46b
I3	68.11 ± 2.25c	1.97 ± 0.05c	4612.6 ± 205.06c	35.07 ± 1.01c	192.45 ± 8.67c
P-value	**	**	**	**	**
LSD_0.05_	*6.98*	*0.18*	*766.49*	*3.03*	*50.14*
**Zeolite**					
Z1	79.56 ± 2.56a	2.34 ± 0.07b	6008.8 ± 317.13b	40.22 ± 1.27b	349.53 ± 29.87b
Z2	92.56 ± 3.20b	2.61 ± 0.09a	7171.7 ± 395.38a	45.66 ± 1.21a	444.94 ± 34.87a
*P-value*	**	*	*	**	*
LSD_0.05_	8.17	0.23	1024.6	3.56	92.94
**Chitosan**					
C1	92.24 ± 5.01a	2.64 ± 0.13a	7270.7 ± 467.40ab	45.37 ± 1.70ab	437.98 ± 49.33a
C2	85.87 ± 4.27ab	2.50 ± 0.10ab	7343.3 ± 549.09a	46.69 ± 1.70a	465.44 ± 51.33a
C3	79.58 ± 3.68b	2.28 ± 0.11b	5832.0 ± 472.04b	40.68 ± 1.76bc	346.57 ± 41.20a
C4	85.91 ± 4.15ab	2.47 ± 0.11ab	5914.8 ± 522.83ab	39.01 ± 1.80c	338.95 ± 41.59a
P-value	ns	ns	ns	**	ns
LSD_0.05_	*12.15*	*0.34*	*1442.2*	*4.99*	*132.06*
Z*C					
Z_1_C1	85.51 ± 5.81a	2.53 ± 0.15a	6639.0 ± 612.56ab	42.67 ± 2.46ab	398.66 ± 63.88a
Z_1_C2	78.80 ± 5.13a	2.31 ± 0.12a	6944.9 ± 742.40a	43.63 ± 2.28a	414.11 ± 6841a
Z_1_C3	74.07 ± 4.92a	2.14 ± 0.015a	5066.0 ± 475.59b	38.85 ± 2.61ab	3.5.72 ± 52.10a
Z_1_C4	79.85 ± 4.37a	2.37 ± 0.14a	5358.1 ± 537.21ab	35.74 ± 2.32b	288.62 ± 52.10a
P-value	ns	ns	ns	ns	ns
LSD_0.05_	*14.79*	*0.42*	*1788*	*7.23*	*177.57*
Z_2_C1	98.97 ± 7.84a	2.74 ± 0.23a	7902 ± 673.41a	48.08 ± 2.11ab	486.3 ± 75.36a
Z_2_C2	92.95 ± 6.22a	2.70 ± 0.16a	7742 ± 831.00a	49.76 ± 2.19a	516.8 ± 76.55a
Z_2_C3	85.09 ± 5.08a	2.57 ± 0.17a	6598 ± 757.71a	42.51 ± 2.35b	387.4 ± 63.90a
Z_2_C4	91.97 ± 6.46a	2.41 ± 0.18a	6445 ± 896.10a	42.28 ± 2.38b	389.3 ± 63.29a
P-value	ns	ns	ns	ns	ns
LSD_0.05_	19.16	0.55	2354.5	6.71	208.91
I*Z					
I1Z_1_	92.85 ± 3.25b	2.72 ± 0.08b	7952.5 ± 290.00b	47.38 ± 1.40b	562.20 ± 29.19b
I1Z_2_	110.36 ± 3.21a	3.19 ± 0.11a	9908.3 ± 306.41a	51.83 ± 0.74a	695.66 ± 24.56a
P-value	**	**	**	*	**
LSD_0.05_	*9.54*	*0.30*	*916.63*	*3.43*	*83.33*
I2Z_1_	81.88 ± 2.47b	2.43 ± 0.05a	5793.6 ± 410.91a	41.54 ± 1.19b	322.71 ± 17.00b
I2Z_2_	94.09 ± 3.28a	2.56 ± 0.09a	6661.8 ± 418.48a	64.74 ± 1.94a	417.93 ± 23.97a
P-value	**	ns	ns	*	**
LSD_0.05_	*8.78*	*0.23*	*1170.3*	*4.93*	*63.64*
I3Z_1_	63.94 ± 2.71b	1.87 ± 0.09b	4280.3 ± 296.36a	31.74 ± 0.89b	163.68 ± 7.78b
I3Z_2_	72.29 ± 3.27a	2.07 ± 0.04a	4944.9 ± 260.65a	38.40 ± 1.21a	221.24 ± 10.18a
P-value	**	*	ns	**	**
LSD_0.05_	5.97	0.16	815.55	3.30	27.98

Means with the same letter in each column are not significantly different at the 5% level based on the LSD test. Slicing was performed based on irrigation regimes and zeolite. I1: 50% ASWD, I2: 65% ASWD, I3: 80% ASWD, Z1: Non-Application of zeolite, Z2: Application of 4.5 t ha^-1^ of zeolite, C1: chitosan 0.4%, C2: chitosan 0.5%, C3: distilled water (control), C4: 1% acetic acid.

In location L2, the trend was similar, with optimal irrigation (I1; 109.52 cm) resulting in the tallest plants, followed by I2 (82.66 cm) and I3 (68.64 cm), showing a 37.14% reduction under severe drought. Zeolite application increased height to 92.28 cm versus 81.60 cm in the control. Among chitosan treatments, 0.5% chitosan (C2) resulted in the greatest height (91.40 cm), with minor differences among other concentrations. Zeolite improved height across all irrigation levels; for instance, under I1, plant height reached 122.88 cm (Z2) compared to 111.99 cm (Z1) Table 4.

**Table 4:** Mean comparison main effects of irrigation regimes (I), zeolite (Z), and chitosan (C), and two-way interactions of I*Z, I*C on sesame traits in Yazd (L2).

Irrigation regimes	Height (cm)	Capsule length (cm)	Biological yield (kg ha^-1^)	Oil yield (kg ha^-1^)
I1	109.52 ± 1.96a	3.07 ± 0.04a	8363.62 ± 181.37a	647.64 ± 15.57a
I2	82.66 ± 1.60b	2.63 ± 0.04b	6044.07 ± 160.30b	426.76 ± 13.92b
I3	68.64 ± 1.16c	1.96 ± 0.03c	4040.59 ± 153.22c	222.38 ± 7.36c
P-value	**	**	**	**
LSD_0.05_	5.46	0.139	552.79	7.38
**Zeolite**				
Z1	81. 60 ± 2.15b	2.42 ± 0.06b	5693.43 ± 229.39b	384.09 ± 20.66b
Z2	92.28 ± 2.47a	2.69 ± 0.06a	6605.42 ± 256.19a	480.43 ± 23.95a
*P-value*	**	**	**	**
*LSD*_*0.05*_	*2.27*	*0.07*	*252.53*	*10.45*
**Chitosan**				
C1	91.40 ± 3.86a	2.70 ± 0.09a	6562.03 ± 363.42a	486.32 ± 33.85b
C2	87.24 ± 3.45b	2.61 ± 0.08ab	6701.70 ± 371.64a	495.92 ± 35.24a
C3	82.48 ± 3.10d	2.38 ± 0.08c	5594.16 ± 311.06b	380.28 ± 28.32c
C4	86.64 ± 3.25c	2.52 ± 0.08b	5739.81 ± 330.78b	384.51 ± 28.96c
P-value	**	**	**	**
LSD_0.05_	3.22	0.09	357.13	14.78
I*Z				
I1Z_1_	111.99 ± 1.31b	3.12 ± 0.05a	7799.47 ± 161.16b	602.22 ± 20.28b
I1Z_2_	122.88 ± 1.18a	3.25 ± 0.06a	8927.76 ± 284.24a	730.51 ± 24.40a
P-value	**	ns	**	**
LSD_0.05_	3.19	0.16	668.18	69.26
I2Z_1_	71.270.065 ± 1.27b	2.55 ± 0.05b	5655.16 ± 205.83b	433.23 ± 15.93b
I2Z_2_	83.40 ± 1.38a	2.97 ± 0.07a	6432.97 ± 222.48a	533.17 ± 13.03a
P-value	**	**	*	**
LSD_0.05_	3.97	0. 15	599.28	45.02
I3Z_1_	67.66 ± 0.99b	1.81 ± 0.03b	3625.67 ± 200.48b	220.51 ± 6.89b
I3Z_2_	70.69 ± 0.59a	2.11 ± 0.04a	4455.51 ± 201.96a	284.10 ± 7.50a
P-value	**	**	**	**
LSD_0.05_	1.84	0.12	571.19	22.13
I*C				
I_1_C_1_	120.86 ± 2.76a	3.34 ± 0.07a	8735.50 ± 390.15ab	713.67 ± 33.69a
I_1_C_2_	119.53 ± 2.49a	3.29 ± 0.06ab	9078.35 ± 325.27a	756.56 ± 33.31a
I_1_C_3_	113.23 ± 3.05a	2.99 ± 0.05c	7737.84 ± 319.39c	590.8 ± 27.93b
I_1_C_4_	166.13 ± 2.62a	3.13 ± 0.09bc	7902.78 ± 297.09bc	604.24 ± 26.71b
P-value	ns	**	*	**
LSD_0.05_	7.98	0.18	970.49	95.50
I2C_1_	81.78 ± 3.26a	2.92 ± 0.10a	6417.10 ± 291.24a	518.79 ± 21.77ab
I2C_2_	77.06 ± 3.26a	2.820.09a	6840.73 ± 211.06a	539.03 ± 25.14a
I2C_3_	74.54 ± 2.80a	2.61 ± 0.12a	5366.88 ± 166.56b	425.92 ± 25.14c
I2C_4_	75.96 ± 2.72a	2.69 ± 0.15a	5551.56 ± 384.66b	449.08 ± 24.34bc
P-value	ns	ns	**	*
LSD_0.05_	9.66	0.33	765.37	71.68
I3C_1_	69.05 ± 0.61a	2.05 ± 0.10a	4533.49 ± 467.14a	263.55 ± 14.83ab
I3C_2_	69.20 ± 1.57a	2.05 ± 0.06a	4186.01 ± 278.29ab	283.64 ± 15.61a
I3C_3_	68.39 ± 1.57a	1.87 ± 0.08a	3677.76 ± 193.70b	225.12 ± 13.57b
I3C_4_	70.04 ± 1.47a	188 ± 0.07a	3765.10 ± 158.76ab	236.91 ± 14.15b
P-value	ns	ns	ns	ns
LSD_0.05_	3.39	0.25	852.94	45.44

Means with the same letter in each column are not significantly different at the 5% level based on the LSD test. Slicing was performed based on irrigation regimes. I1: 50% ASWD, I2: 65% ASWD, I3: 80% ASWD, Z1: Non-Application of zeolite, Z2: Application of 4.5 t ha^-1^ of zeolite, C1: chitosan 0.4%, C2: chitosan 0.5%, C3: distilled water (control), C4: 1% acetic acid.

#### 3.1.2. Capsule length.

**Fig 3:**
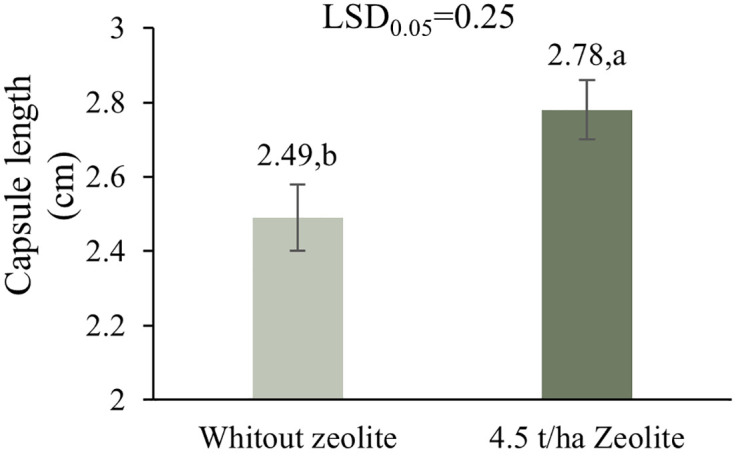
Comparison of the means of main effect of zeolite on the Capsule length in L2.

In L1, optimal irrigation (I1) produced the longest capsules (2.96 cm), while moderate (I2) and severe stress (I3) reduced lengths by 15.88% and 33.45%, respectively. Zeolite (Z2) increased capsule length by 11.54% over control (2.61 vs. 2.34 cm). Chitosan at 0.4% (C1) achieved the highest capsule length (2.64 cm), whereas other treatments (C2–C4) showed reduced values Table 3. At L2, C1 had the highest capsule length (2.70 cm), with C2 (2.61 cm), C4 (2.52 cm), and C3 (2.38 cm) following. The interaction of irrigation and zeolite (I\*Z) showed that under I1, Z2 improved capsule length to 3.25 cm (4.17% more than Z1). Under I2 and I3, Z2 increased lengths by 16.47% and 16.57% over the control, respectively Table 4. In L2 Application of 4.5 t/ha zeolite significantly increased capsule length by 11.65% (from 2.49 cm to 2.78 cm) compared to the control without zeolite (Fig 3).

#### 3.1.3. Leaf area index (LAI).

**Fig 4:**
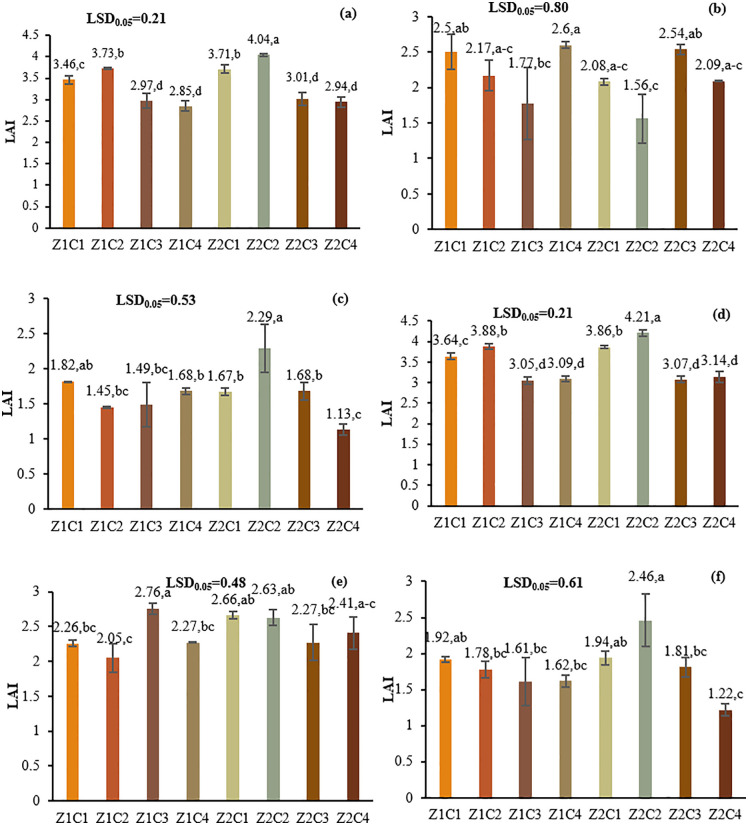
Comparison of the means of the four-way interaction effect L*I*Z*C on the leaf area index (LAI). Slicing was performed based on location and irrigation regimes. Means with the same letter in each column, based on the LSD test, do not show a significant difference at the 5% level. (a): L1, Optimal irrigation; (b): L1, Moderate water deficit stress; (c): L1, Severe water deficit stress; (d): L2, Optimal irrigation; (e): L2, Moderate water deficit stress; (f): L2, Severe water deficit stress. L1: Tehran, L2: Yazd, I1: 50% ASWD, I2: 65% ASWD, I3: 80% ASWD, Z1C1: Non-Application of zeolite+ chitosan 0.4%, Z1C2: Non-Application of zeolite+ chitosan 0.5%, Z1C3: Non-Application of zeolite+ distilled water (control), Z1C4: Non-Application of zeolite+1% acetic acid, Z2C1: Application of 4.5 t ha⁻^1^ of zeolite + chitosan 0.4%, Z2C2: Application of 4.5 t ha⁻^1^ of zeolite + chitosan 0.5%, Z2C3: Application of 4.5 t ha^-1^ of zeolite + distilled water (control), Z2C4: Application of 4.5 t ha⁻^1^ of zeolite +1% acetic acid.

LAI was significantly affected by the L*I*Z*C interaction. Under optimal irrigation, Z2C2 treatments had the highest LAI values: 4.04 in L1 and 4.21 in L2, showing 36–38% increases over controls. Under mild stress, combinations like Z1C4 (L1; 2.6) and Z1C3 (L2; 2.76) performed best. Under severe stress, Z2C2 showed the highest LAI: 2.29 in L1 and 2.46 in L2, indicating effective mitigation of drought effects through zeolite and chitosan applications (Fig 4).

### 3.2. Yield and yield components

#### 3.2.1. Number of grains per capsule.

**Fig 5:**
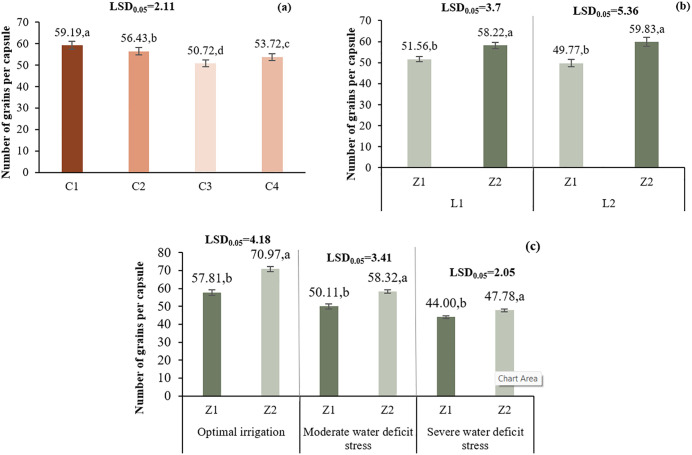
Comparison of the mean main effect of C (a) and the two-way interaction effects of L*Z (b) and I*Z (c) on the number of grains per capsule. Means with the same letter in each column, based on the LSD test, do not show a significant difference at the 5% level. L1: Tehran, L2: Yazd, I1: 50% ASWD, I2: 65% ASWD, I3: 80% ASWD, Z1: Non-Application of zeolite, Z2: Application of 4 5 t ha-1 of zeolite, C1: chitosan 0.4%, C2: chitosan 0.5%, C3: distilled water (control), C4: 1% acetic acid.

Zeolite (Z2) under I1 resulted in the highest number of grains per capsule (70.97), a 22.7% increase over control. Similar increases were observed under I2 (16.38%) and I3 (8.59%). In L2, Z2 outperformed Z1 (59.83 vs. 49.77), a 20.2% increase. At L1, Z2 also improved grain number by 12.9%. Chitosan at 0.4% (C1) yielded the highest grain number (59.19), followed by C2 and C4 (Fig 5).

#### 3.2.2. 1000-seed weight.

**Fig 6:**
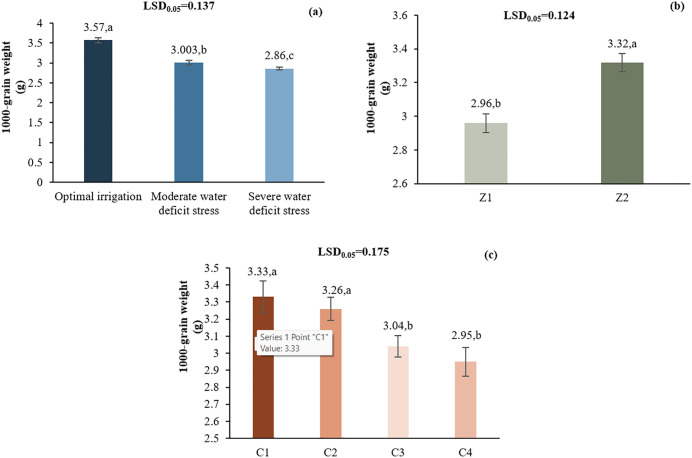
Comparison of the mean main effects of I (a), Z (b), and C (c) on the 1000-grain weight. Means with the same letter in each column, based on the LSD test, do not show a significant difference at the 5% level. L1: Tehran, L2: Yazd, I1: 50% ASWD, I2: 65% ASWD, I3: 80% ASWD, Z1: Non-Application of zeolite, Z2: Application of 4.5 t ha-1 of zeolite, C1: chitosan 0.4%, C2: chitosan 0.5%, C3: distilled water (control), C4: 1% acetic acid.

Optimal irrigation resulted in the highest 1000-grain weight (3.57 g). I2 and I3 caused 15.77% and 19.88% decreases, respectively. Zeolite (Z2) increased grain weight by 12.2% compared to Z1 (3.32 vs. 2.96 g). C1 had the highest grain weight (3.33 g), followed by C2 (3.26 g), C3 (3.04 g), and C4 (2.95 g) (Fig 6).

#### 3.2.3. Grain yield.

**Fig 7:**
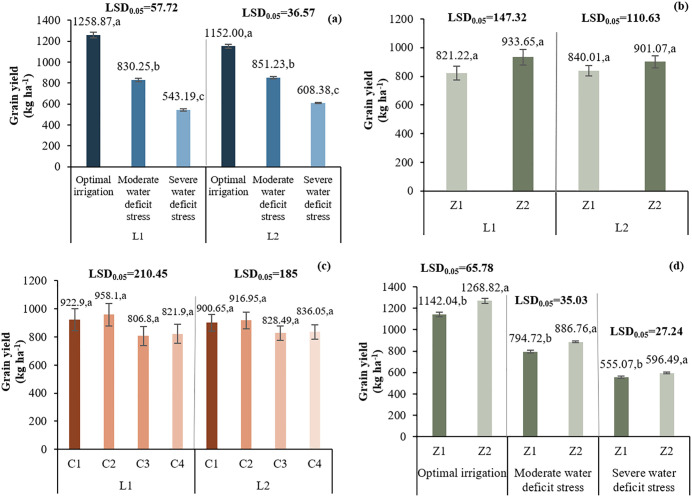
Comparison of the mean two-way interaction effects of L*I (a), L*Z (b), L*I (c), and I*Z (d) on grain yield. Means with the same letter in each column, based on the LSD test, do not show a significant difference at the 5% level. L1: Tehran, L2: Yazd, I1: 50% ASWD, I2: 65% ASWD, I3: 80% ASWD, Z1: Non-Application of zeolite, Z2: Application of 4.5 t ha-1 of zeolite, C1: chitosan 0.4%, C2: chitosan 0.5%, C3: distilled water (control), C4: 1% acetic acid.

In L1, I1 led to the highest yield (1258.87 kg ha^-1^). I3 caused a 56.85% yield reduction. In L2, I2 and I3 decreased yields by 26.10% and 47.18%, respectively. Z2 increased yield in both locations: L1 (933.65 vs. 821.22 kg ha^-1^) and L2 (901.07 vs. 840.01 kg ha^-1^). Chitosan C2 produced the highest yields in both locations. I*Z interaction showed Z2 under I1 had the highest yield (1268.82 kg ha^-1^), with increases of 11.1% (I1), 11.58% (I2), and 7.5% (I3) compared to Z1(Fig 7).

#### 3.2.4 Biological yield.

**Fig 8:**
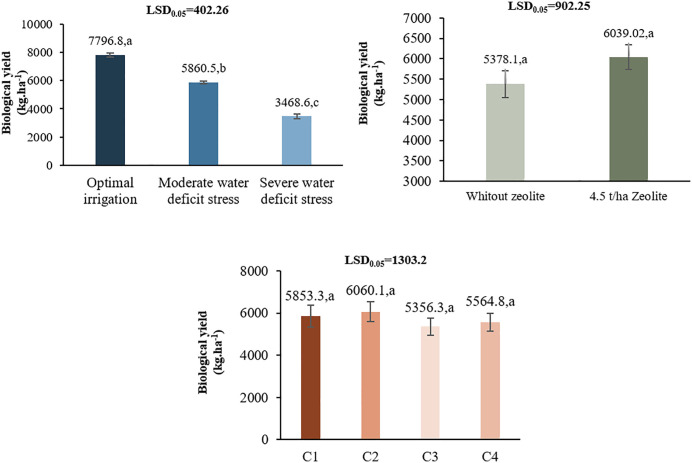
Comparison of the means of main effects of irrigation regimes, zeolite and chitosan on the biological yield in L2.

At L1, C2 treatment had the highest biological yield (7343.3 kg ha^-1^). Z2 under I1 produced 9908.3 kg ha^-1^, a 24.58% increase over control. Under I2 and I3, Z2 also increased yields by 14.96% and 15.53%, respectively Table 3. At L2, I1 gave the highest biological yield (9908.3 kg ha^-1^), while Z2 improved yields by 19.33% over Z1. Among chitosan treatments, C2 again performed best Table 4. At L2 Moderate and severe water deficit stress reduced biological yield by 24.6% and 55.5%, respectively, compared to optimal irrigation. Application of 4.5 t/ha zeolite significantly increased biological yield by 12.3% compared to the control without zeolite. Compared to the distilled water control (5356.3 kg/ha), 0.4% and 0.5% chitosan increased biological yield by 9.3% and 13.1% respectively, while 1% acetic acid increased it by 3.9% (Fig 8).

#### 3.2.5. Harvest index.

**Fig 9:**
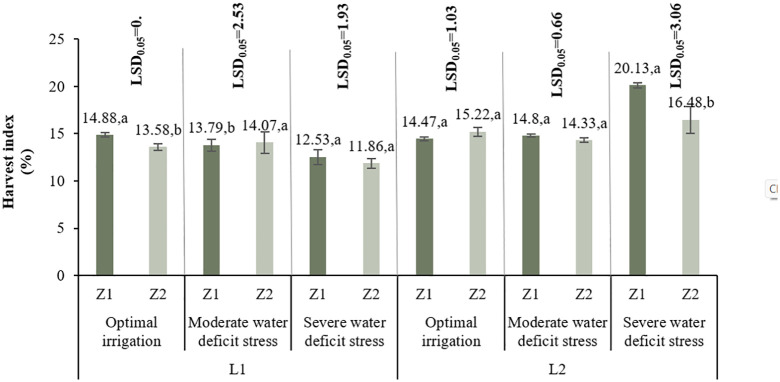
Comparison of the mean triple-way interaction effect of L*I*Z on the Harvest Index. Means with the same letter in each column, based on the LSD test, do not show a significant difference at the 5% level. L1: Tehran, L2: Yazd, I1: 50% ASWD, I2: 65% ASWD, I3: 80% ASWD, Z1: Non-Application of zeolite, Z2: Application of 4.5 t ha-1 of zeolite.

In L1, under I1, Z1 had the highest harvest index (14.88%). Z2 decreased it by 8.73%. Under I2, Z2 increased the index by 2.03%, while under I3, it decreased by 5.35%. In L2, Z2 improved harvest index under I1 by 5.18% but reduced it significantly under I3 (22.1% reduction), indicating location-specific responses (Fig 9).

#### 3.2.6. Water use efficiency (WUE).

**Fig 10:**
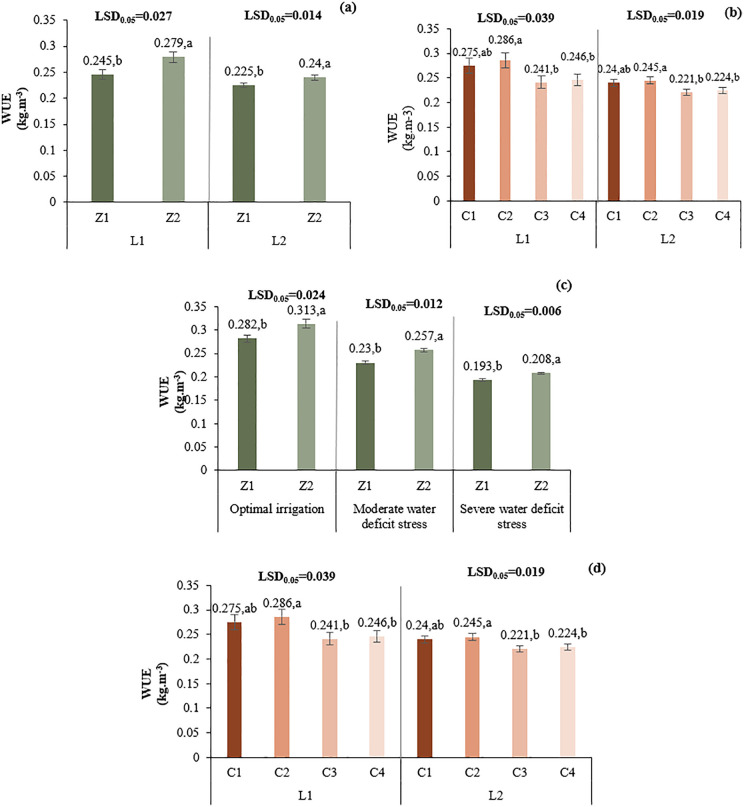
Comparison of the mean two-way interaction effects of L*Z (a), L*C (b), I*Z (c) I*C (d) on Water use efficiency. Means with the same letter in each column, based on the LSD test, do not show a significant difference at the 5% level. L1: Tehran, L2: Yazd, I1: 50% ASWD, I2: 65% ASWD, I3: 80% ASWD, Z1: Non-Application of zeolite, Z2: Application of 4.5 t ha-1 of zeolite, C1: chitosan 0.4%, C2: chitosan 0.5%, C3: distilled water (control), C4: 1% acetic acid.

At L1 (Tehran), Z2 increased water use efficiency (WUE) by 13.9% over Z1, while in L2 (Yazd), the increase was 6.7%, highlighting a stronger zeolite effect in the cooler site (Fig 10). In both locations, C2 (chitosan 0.5%) resulted in the highest WUE, with increases of 4.0% over the control (C3) in L1 and 10.9% in L2, whereas C4 (1% acetic acid) showed minimal improvement. Across irrigation regimes, Z2 consistently enhanced WUE compared to Z1: by 11.0% under optimal irrigation (80% ASWD), 11.7% under moderate water deficit stress (65% ASWD), and 7.8% under severe water deficit stress (50% ASWD), demonstrating zeolite’s mitigating role in drought conditions. The Z2 and C2 combination exhibited the most notable increases in WUE across all irrigation levels, with C2 significantly improving WUE under both Z1 and Z2, particularly in moderate to severe stress where antioxidant and water retention benefits were amplified (Fig 10).

### 3.3. Oil content and oil yield

#### 3.3.1. Oil content.

**Fig 11:**
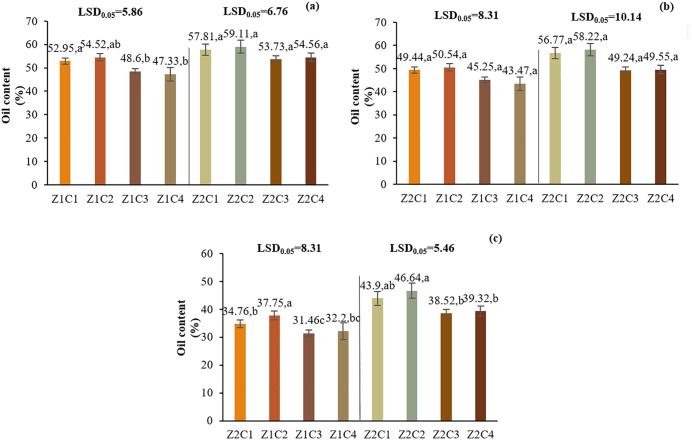
Comparison of the mean triple-way interaction effect of I*Z*C on oil content for Yazd L2. (a): Optimal irrigation, (b): Moderate water deficit stress, (c): Severe water deficit stress. Means with the same letter in each column, based on the LSD test, do not show a significant difference at the 5% level. Z1: Non-Application of zeolite, Z2: Application of 4.5 t ha-1 of zeolite, C1: chitosan 0.4%, C2: chitosan 0.5%, C3: distilled water (control), C4: 1% acetic acid.

In L1, I1 had the highest oil content (49.60%), while I2 and I3 reduced it by 11.01% and 29.29%, respectively. Z2 increased oil content by 13.55%. C2 (0.5% chitosan) had the highest oil content (46.69%), followed by C1, C3, and C4 (Table 3). In L2, the triple interaction (I*Z*C) showed that Z2C2 had the highest oil content (59.11%) under I1. Similar trends were observed under I2 and I3 (Fig 11).

#### 3.3.2. Oil yield.

In L1, Z2 under I1 increased oil yield by 23.71% (695.66 vs. 562.20 kg/ha). Under I2 and I3, Z2 improved yields by 29.49% and 35.16%. Chitosan C2 had the highest oil yields under both Z1 and Z2 conditions Table 3. In L2, Z2 consistently outperformed Z1 under all irrigation regimes. C2 also provided the highest oil yields under all irrigation levels Table 4.

### 3.4. Biochemical traits

#### 3.4.1. Malondialdehyde (MDA).

**Fig 12:**
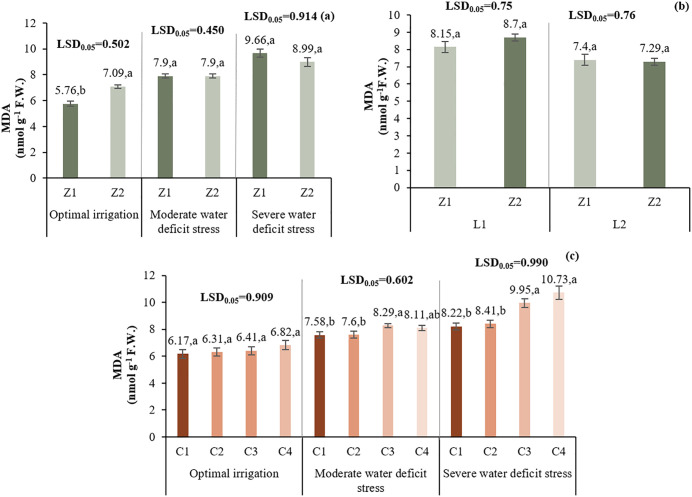
Comparison of the mean two-way interaction effects of I*Z (a), L*Z (b), I*C (c) on malondialdehyde. Means with the same letter in each column, based on the LSD test, do not show a significant difference at the 5% level. L1: Tehran, L2: Yazd, I1: 50% ASWD, I2: 65% ASWD, I3: 80% ASWD, Z1: Non-Application of zeolite, Z2: Application of 4.5 t ha-1 of zeolite, C1: chitosan 0.4%, C2: chitosan 0.5%, C3: distilled water (control), C4: 1% acetic acid.

Under I1, Z2 increased MDA by 23% compared to Z1. No difference was observed under I2. Under I3, Z2 decreased MDA by 6.9%. In L1, Z2 increased MDA by 6.7%, while in L2, it decreased by 1.5%. C1 treatment resulted in the lowest MDA across all stress levels, while C4 and C3 showed higher MDA values (Fig 12).

#### 3.4.2 Soluble protein.

**Fig 13:**
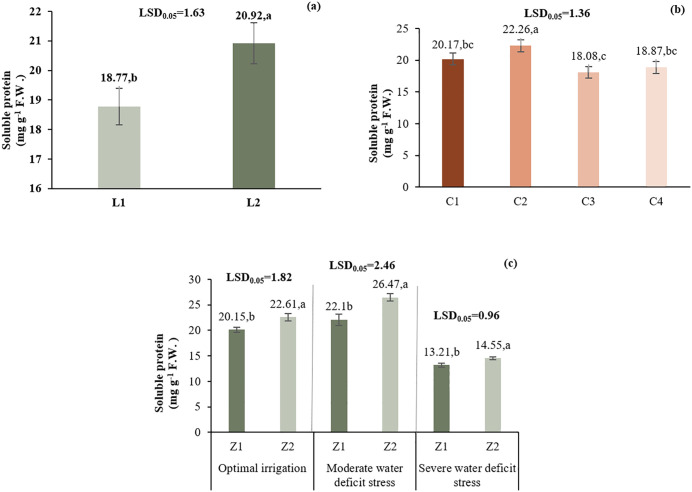
Comparison of the mean main effect of L (a), C (b) and the two-way interaction effects of I*Z (c) on the soluble protein. Means with the same letter in each column, based on the LSD test, do not show a significant difference at the 5% level. L1: Tehran, L2: Yazd, I1: 50% ASWD, I2: 65% ASWD, I3: 80% ASWD, Z1: Non-Application of zeolite, Z2: Application of 4.5 t ha-1 of zeolite, C1: chitosan 0.4%, C2: chitosan 0.5%, C3: distilled water (control), C4: 1% acetic acid.

L2 showed higher soluble protein levels (20.92 mg g ⁻ ¹FW) than L1 (18.77 mg g ⁻ ¹FW). C2 had the highest protein content (22.26 mg g ⁻ ¹FW), followed by C1, C4, and C3. Z2 under I1 had the highest protein content (26.47 mg g ⁻ ¹FW), with increases under I2 (19.7%) and I3 (9.89%) as well (Fig 13).

#### 3.4.3. Peroxidase (POD).

**Fig 14:**
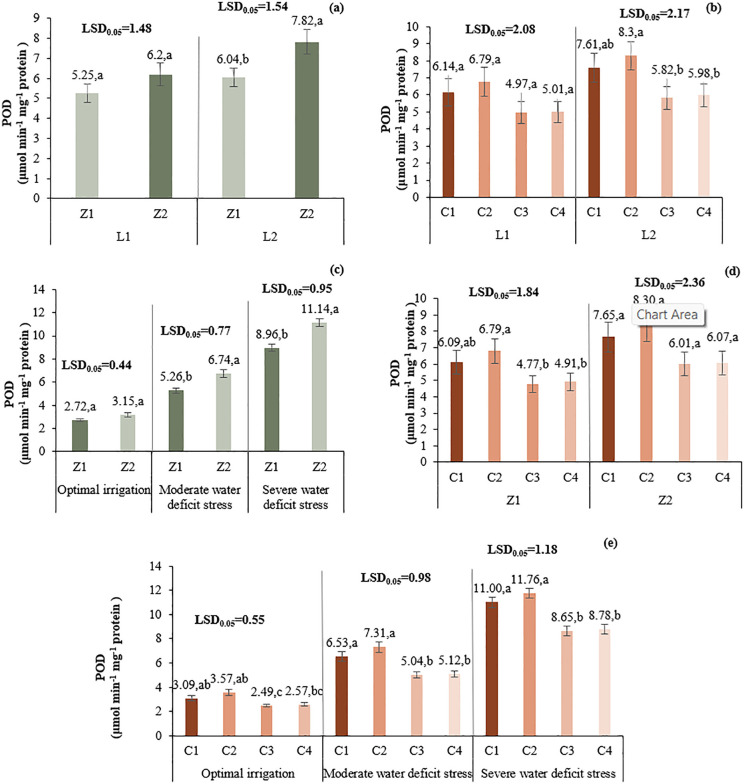
Comparison of the mean two-way interaction effects of L*Z (a), L*C (b), I*Z (c), Z*C (d) and I*C (e) on peroxidase activity. Means with the same letter in each column, based on the LSD test, do not show a significant difference at the 5% level. L1: Tehran, L2: Yazd, I1: 50% ASWD, I2: 65% ASWD, I3: 80% ASWD, Z1: Non-Application of zeolite, Z2: Application of 4.5 t ha-1 of zeolite, C1: chitosan 0.4%, C2: chitosan 0.5%, C3: distilled water (control), C4: 1% acetic acid.

At L1, Z2 increased POD activity by 18.1% over Z1. In L2, the increase was 29.5%. C2 resulted in the highest POD activity in both locations. Z2 and C2 combination showed the most notable increases across all irrigation levels, with C2 significantly improving POD under both Z1 and Z2 (Fig 14).

#### 3.4.4. Catalase (CAT).

**Fig 15:**
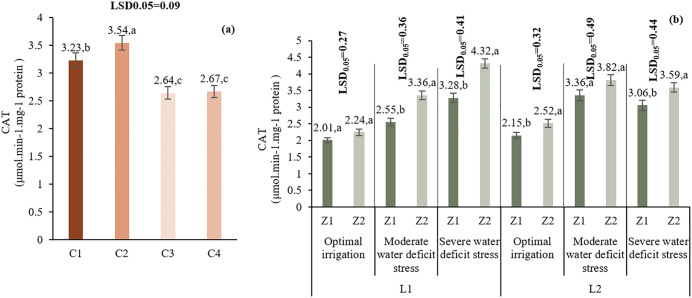
Comparison of the mean main effect of C (a) and the triple-way interaction effect of L*I*Z (b) on catalase activity. Means with the same letter in each column, based on the LSD test, do not show a significant difference at the 5%. I1: 50% ASWD, I2: 65% ASWD, I3: 80% ASWD, Z1: Non-Application of zeolite, Z2: Application of 4.5 t ha-1 of zeolite, C1: chitosan 0.4%, C2: chitosan 0.5%, C3: distilled water (control), C4: 1% acetic acid.

C2 treatment had the highest CAT activity (3.54 µmol H₂O₂ min⁻^1^mg⁻^1^ protein), followed by C1, C4, and C3. The L*I*Z interaction showed that in L1 under I1, Z2 significantly increased CAT activity. Under I3, Z1 performed better. In L2, Z2 had higher CAT under I1, while Z1 had better results under drought (Fig 15).

### 3.5. Correlation between traits

**Fig 16:**
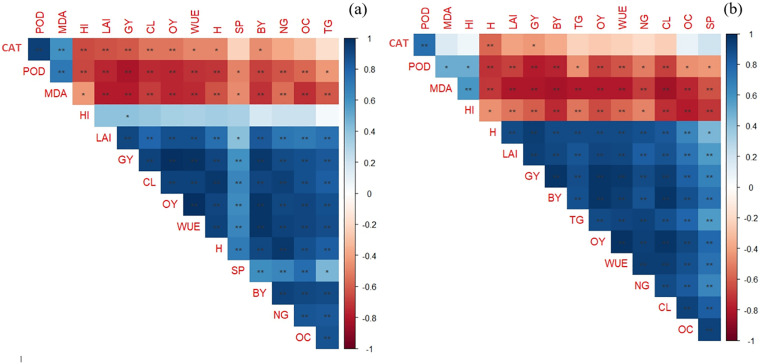
Correlation coefficients between sesame traits. a: Location 1 (Tehran), b: Location 2 (Yazd). The color spectrum, from dark blue to dark red, represents correlations ranging from highly positive to highly negative. Stars indicate the significance of correlations (* P ≤ 0.05,** P ≤ 0.01). H: Height, CL: Capsule length, NG: Number of grains per capsule, TG: 1000-grain weight, BY: Biological yield, GY: Grain yield, OC: Oil content, OY:Oil yield, HI: Harvest index, MDA: Malondialdehyde, SP: Soluble protein, POD: Peroxidase activity, CAT: Catalase activity and WUE: Water use efficiency.

Significant correlations (P ≤ 0.01) were observed among several physiological and agronomic traits. A strong positive correlation (r = 0.6–0.8) was found between peroxidase (POD) activity and malondialdehyde (MDA) content, indicating that higher oxidative stress is associated with increased antioxidant enzyme activity. The harvest index showed moderate to strong positive correlations (r = 0.4–0.7) with both grain yield and biological yield, suggesting that improvements in resource allocation efficiency contribute to higher productivity (Fig 16). Interestingly, catalase (CAT) activity was negatively correlated with oil content (r = −0.6 to −0.8), implying a potential physiological trade-off between stress response and oil accumulation. In L2, oil content exhibited strong negative correlations with capsule length and the number of grains per capsule, reflecting environmental influences that may hinder simultaneous enhancement of both yield components and oil quality. These findings highlight the complex interactions between physiological responses and yield-related traits under different environmental and management conditions, reinforcing the need for integrated, location-specific optimization strategies in sesame cultivation (Fig 16).

### 3.6. Principal component analysis

**Fig 17:**
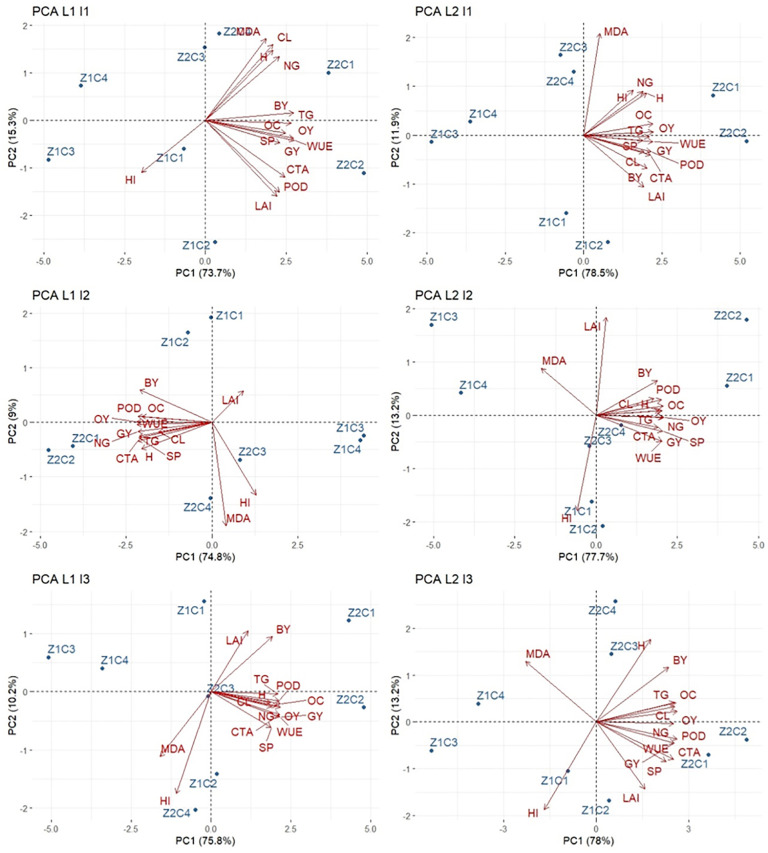
Principal component analysis (PCA) biplot of traits in sesame as affected by zeolite and chitosan-based treatments under three irrigation regimes at two locations (Tehran and Yazd, Iran). H: Height, CL: Capsule length, NG: Number of grains per capsule, TG: 1000-grain weight, BY: Biological yield, GY: Grain yield, OC: Oil content, OY:Oil yield, HI: Harvest index, MDA: Malondialdehyde, SP: Soluble protein, POD: Peroxidase activity and CAT: Catalase activity WUE: Water use efficiency.

Principal component analysis (PCA) was applied to comprehensively assess the multivariate relationships among morphological, physiological, biochemical, and yield-related traits of sesame subjected to different irrigation regimes and zeolite–chitosan-based treatments at two contrasting locations, Tehran (L1) and Yazd (L2) (Fig. 17). The PCA biplots provided a clear visualization of treatment discrimination and trait interrelationships, allowing identification of dominant response patterns under varying environmental and water availability conditions.

Across all irrigation regimes and locations, the first two principal components accounted for a high proportion of the total variance, indicating the robustness and explanatory power of the PCA model. The first principal component (PC1) explained between 73.7% and 78.5% of the total variation, while the second principal component (PC2) contributed 10.2% to 15.3%, resulting in a cumulative variance exceeding 85% in all cases. This substantial explained variance demonstrates that the major sources of variability in sesame responses were effectively captured by PC1 and PC2.

PC1 was consistently and positively associated with key growth, productivity, and physiological efficiency traits, including leaf area index (LAI), grain yield (GY), biological yield (BY), oil yield (OY), oil content (OC), thousand-grain weight (TG), number of grains per capsule (NG), capsule length (CL), water use efficiency (WUE), soluble protein (SP), and the antioxidant enzymes catalase (CAT) and peroxidase (POD). The close proximity and similar orientation of these trait vectors indicate strong positive interrelationships, showing that improved morphological development, physiological performance, and antioxidant capacity were closely associated with enhanced yield and oil production. In contrast, malondialdehyde (MDA), a marker of lipid peroxidation and oxidative damage, was consistently positioned in the opposite direction of PC1 and showed negative associations with yield- and growth-related traits. Harvest index (HI) also tended to align away from the positive PC1 axis, particularly under water-limited conditions, indicating altered biomass partitioning under stress.

Under optimal irrigation (I1) in Tehran (L1), non-zeolite treatments were generally positioned closer to the positive side of PC1 and were associated with higher values of LAI, GY, SP, CAT, and POD. In contrast, several zeolite-based treatments were located farther from the main cluster of productivity-related traits, indicating a weaker association with growth and antioxidant attributes under non-limiting water conditions. In Yazd (L2), however, even under optimal irrigation, zeolite–chitosan treatments exhibited a closer association with favorable traits. Treatments such as Z2C1 and Z2C3 were positioned near the vectors representing yield, oil traits, antioxidant enzymes, and WUE, while being oriented opposite to MDA, indicating a location-dependent response likely influenced by higher temperature and evaporative demand.

Under moderate drought stress (I2), a more distinct separation between productive and stress-related traits was observed at both locations. PC1 continued to be dominated by yield components, oil traits, WUE, SP, and antioxidant enzyme activities, emphasizing the central role of these traits in stress adaptation. In Tehran (L1), zeolite–chitosan treatments (particularly Z2C1 and Z2C2) were grouped in the positive quadrant of the biplot and showed strong associations with PC1-related traits, while non-zeolite treatments were positioned closer to the neutral or negative regions of PC1. In Yazd (L2), a similar but more pronounced pattern was observed, with zeolite-based treatments consistently aligned with productivity- and defense-related traits and clearly separated from MDA along both PC1 and PC2.

Under severe drought stress (I3), the PCA structure highlighted a pronounced contrast between stress-tolerant and stress-sensitive trait combinations. PC1 (explaining up to 78.0% of the variance) remained strongly associated with GY, OY, BY, OC, WUE, SP, CAT, and POD, indicating that the maintenance of antioxidant defense, protein stability, and efficient water use was closely linked to yield preservation under severe water deficit. Across both locations, zeolite–chitosan treatments were consistently positioned closer to the positive PC1 axis, whereas MDA and HI were oriented in the opposite direction, reflecting increased oxidative damage and altered assimilate allocation under drought.

Although the overall PCA patterns were consistent across locations, the separation of treatments along PC2 revealed location-specific modulation of stress responses. The greater dispersion of treatments along PC2 in Yazd suggests enhanced sensitivity of biochemical and oxidative stress indicators to environmental conditions in the warmer and drier region, likely arising from interactions between irrigation regime, soil amendment efficacy, and local climatic factors.

Overall, PCA clearly distinguished productive, stress-tolerant trait complexes from stress-associated traits across irrigation regimes and locations. The strong positive associations among yield components, oil traits, antioxidant enzyme activities, soluble protein, and water use efficiency indicate that these traits form an integrated response network contributing to drought tolerance in sesame. Conversely, the consistent negative association of MDA with these traits confirms its effectiveness as a biochemical indicator of drought-induced oxidative stress. The PCA results further demonstrate that the effectiveness of zeolite–chitosan treatments is dependent on both irrigation regime and environmental conditions, with more pronounced associations under moderate to severe water stress and in the warmer Yazd region.

## 4. Discussion

Optimal irrigation results in the highest plant height due to enhanced cell elongation and plant growth processes. Water availability plays a crucial role in tissue expansion, which is tightly coordinated with cell wall mechanical properties and plant hydraulics [29]. Moderate water deficit stress can improve root growth, leading to increased cytokinin concentrations, leaf photosynthetic rates, and grain yield compared to severe water stress or continuous flooding. However, severe water deficits can result in stunted growth and poor establishment [30]. Zeolite application can significantly increase plant height and overall growth in crops. This effect is attributed to zeolite’s ability to improve soil properties, enhance nutrient availability, and increase water retention. Studies have shown that zeolite application rates of 5–7.5 t ha ⁻ ¹ can lead to increased plant height, grain yield, and water use efficiency [31]. Zeolite application has been found to soil characteristics such as cation exchange capacity and pH [32]. chitosan application has shown varied effects on plant growth across different studies. In sweet basil, 4 ml/L chitosan applied during the vegetative stage significantly increased plant height and other growth parameters [33]. The interaction of irrigation and zeolite can significantly improve plant growth and yield under various water regimes [34,35]. for lavender, the combination of 80% irrigation rate with zeolite and lithovit resulted in the highest vegetative growth and oil yield [36]. Drought stress significantly reduces plant height and stem elongation through various physiological mechanisms. It decreases CO2 assimilation and stomatal conductance, leading to reduced photosynthesis and overall growth [37,38]. At the cellular level, drought stress alters gibberellin-related gene expression, particularly downregulating SlGA20ox4, SlGA2ox5, and LeEXP1, which are involved in cell expansion [39]. The stress-induced generation of reactive oxygen species further impacts cellular metabolism [37,38]. Zeolite improved soil properties like bulk density, porosity, and water-holding capacity, allowing plants to access water more efficiently [34,35]. Optimal irrigation generally produces longer capsules and higher seed set in plants like Eucalyptus globulus and capsicum species. However, excessive irrigation can lead to increased vegetative growth at the expense of reproductive structures [40] Water availability significantly influences capsule development and elongation, with moderate deficit irrigation (90% of irrigation water requirement) often yielding the best results in terms of fruit size, weight, and quality [41]. In hemp, zeolite application reduced the negative impact of water deficit on dry matter and oil yield, especially under mild stress conditions [42]. Similarly, in canola, zeolite application significantly increased seed yield and yield components under drought stress [43]. The beneficial effects of zeolite are attributed to its ability to improve soil water status, reduce nitrogen leaching, and protect ammonium against nitrification [44,45]. in okra, foliar application of chitosan at 100−125 ppm resulted in increased morphological, growth, and biochemical parameters, leading to a 27.9% increase in fruit yield [46]. Chitosan-based biostimulants have also been found to enhance growth and nutraceutical properties in *Capsicum chinensis* L., increasing fruit weight and secondary metabolite production [47]. The combined application of zeolite and chitosan can enhance leaf expansion and canopy development in crops under various irrigation conditions. Chitosan has been shown to improve plant growth, biomass production, and water stress tolerance when applied as a foliar spray it can increase leaf area, relative chlorophyll content, and water use efficiency in plants subjected to deficit irrigation [48]. The combination of optimal irrigation and biostimulants like chitosan can significantly increase leaf area index (LAI) and overall plant growth [49]. Furthermore, the application of biostimulants in conjunction with regulated deficit irrigation can enhance macronutrient uptake, yield, and water use efficiency in crops [50]. Chitosan nanoparticles have been shown to mitigate drought stress effects in various crops. In wheat, chitosan application increased leaf area, relative water content, and photosynthesis rate under drought conditions [51]. Chitosan application in thyme plants under drought stress increased nutrient accumulation and altered phenolic composition, with cinnamic acid and rosmarinic acid contents increasing significantly [52]. chitosan coating enhanced wheat seedling growth, antioxidant enzyme activities, and chlorophyll content under drought stress [53]. while chitosan enhances antioxidant enzyme activities, reduces lipid peroxidation, and acts as an antitranspirant [53,54]. Zeolite and zinc applications can significantly improve canola yield and quality under both optimal and drought stress conditions. Under optimal irrigation (I1), zeolite application (Z2) at 15 t ha-1 combined with 0.1% zinc sulfate foliar spray (Zn2) resulted in the highest grain yield, biological yield, and harvest index (Shahsavari et al., 2014). This combination enhanced reproductive development, increasing grain number per capsule compared to no zeolite application (Z1) [55]. Similarly, zeolite application enhanced wheat grain yield and improved soil chemical properties in Iraq [56]. Chitosan’s beneficial effects on plant growth and yield are attributed to its ability to regulate stomatal closure, enhance photosynthesis, and increase the production of key enzymes and plant hormones [57]. Acetic acid treatment has been shown to improve drought tolerance in various plants, including soybean and cassava, by enhancing photosynthesis, osmoregulation, mineral uptake, and antioxidant defense mechanisms [58]. In soybean, acetic acid application resulted in increased root biomass, leaf area, and water use efficiency, while reducing oxidative stress [59]. Similarly, in cassava, acetic acid treatment led to higher leaf relative water content and increased expression of ABA signaling-related genes, enhancing drought avoidance [58]. Water stress decreases biomass in *Dracocephalum kotschyi* [60], fodder protein in Persian clover [61], oil yield, photosynthesis rate, and grain yield in oilseed rape [62–64], quantum efficiency of Photosystem II in European borage [65], grain yield in camelina [66,67], and oil percentage in soybean [68]. Additionally, it increases water-soluble carbohydrate and neutral detergent fiber in *Dracocephalum kotschyi* [69]. For wheat, supplementary irrigation at critical growth stages, such as heading and anthesis, can significantly improve grain yield and quality [70]. Zeolite improves agronomic and biochemical traits, increasing grain yield by 20.1% and 61.9% under drought stress, suggesting enhanced water use efficiency in maize production [71]. Chitosan reduces transpiration, improving water use efficiency without affecting dry matter production in drought-stressed African marigold plants [72]. Chitosan enhances water use efficiency in drought-stressed Piper longum plants, particularly at a concentration of 1 g L⁻^1^ [73].

## 5. Conclusion

Zeolites promote nutrient and water uptake by increasing soil porosity, while chitosan, as a biodegradable and environmentally friendly biopolymer, stimulates plant metabolic activity and facilitates the efficient movement of active compounds across plant cell membranes. Therefore, the overall effect of these products is expected to improve drought tolerance and enhance sesame growth and yield under water stress conditions. This study demonstrated that zeolite (4.5 t ha⁻^1^) and chitosan (0.5%) alleviated the negative impacts of water deficit stress on sesame, thereby supporting sustainable production by achieving maximum grain yield and oil content while minimizing water use. Zeolite increased grain yield under moderate water stress by up to 11.58% and oil yield under severe water stress by 35.16%, whereas chitosan (C2) increased grain yield by 18.8% in L1 and oil content by 59.11% in L2. Moreover, zeolite and chitosan enhanced catalase and peroxidase activities by up to 42.6% and 29.5%, respectively, indicating a reduction in oxidative stress. Thus, zeolite improved soil moisture retention for the sesame crop, while chitosan strengthened physiological defense mechanisms to achieve high yields under water-limiting conditions. However, the study also revealed variations in the effectiveness of zeolite and chitosan across different stress levels and sites. Optimal results were achieved under moderate stress with the combined application of zeolite and chitosan (Z2C2). These findings suggest that zeolite and chitosan are valuable tools and effective strategies to enhance drought resistance in sesame, thereby supporting sustainable agriculture in water-limited regions. Overall, this approach offers an effective means to mitigate drought stress, protect sesame plants against adverse environmental conditions, promote organic farming, and improve understanding of how organic compounds enhance plant drought tolerance.

## Supporting information

S1 File The original data of this article are presented in the supporting file. (RAR)
